# Challenges Encountered During the Veterinary Disaster Response: An Example from Chile

**DOI:** 10.3390/ani3041073

**Published:** 2013-11-21

**Authors:** Elena Garde, Guillermo Enrique Pérez, Gerardo Acosta-Jamett, Barend Mark Bronsvoort

**Affiliations:** 1 Division of Pathway Medicine, School of Biomedical Sciences, College of Medicine and Veterinary Medicine, The University of Edinburgh, Edinburgh, EH16 4SB, UK; 2 Latin America Branch, Veterinarians Without Borders (Veterinarios Sin Fronteras) Canada, Pasaje Los Arrayanes 333, Valdivia, Chile; E-Mail: guillermo@vetswithoutborders.ca; 3 Instituto de Medicina Preventiva Veterinaria, Facultad de Ciencias Veterinarias, Universidad Austral de Chile, Casilla 567, Valdivia, Chile; E-Mail: gerardo.acosta@uach.cl; 4 The Roslin Institute and Royal (Dick) School of Veterinary Studies, University of Edinburgh, Midlothian, EH25 9RG, UK; E-Mail: mark.bronsvoort@roslin.ed.ac.uk

**Keywords:** disaster preparedness, contingency planning, companion animals, earthquake, tsunami, free-roaming dogs, natural disaster, disaster research, disaster response

## Abstract

**Simple Summary:**

Disaster preparedness for companion animals has economic, social and welfare benefits, yet many countries continue to omit dogs and cats from their national and regional contingency planning. Responses therefore, are often chaotic, inefficient and uncoordinated, or absent altogether. Documented experiences in Chile contribute to the information supporting the inclusion of companion animals into locally relevant disaster plans. These plans serve to prepare communities and authorities, identify resources available, establish a chain of command, develop local priorities, and subsequently reduce the negative impacts on both human and animal communities.

**Abstract:**

Large-scale disasters have immeasurable effects on human and animal communities. Evaluating and reporting on the response successes and difficulties encountered serves to improve existing preparedness documents and provide support to those in the process of developing plans. Although the majority of disasters occur in low and middle income nations, less than 1% of the disaster literature originates from these countries. This report describes a response to a disease outbreak in domestic dogs in Dichato, Chile following the 2010 earthquake/tsunami. With no national plan coordinating the companion animal response, there was a chaotic approach among animal welfare organizations towards rescue, diagnosis, treatment and record-keeping. Similar to the medical response following the 1985 earthquake near Santiago, we experienced problems within our own teams in maintenance of data integrity and protocol compliance. Loss of infrastructure added complications with transportation, communications and acquisition of supplies. Similar challenges likely occur in most disasters, but can be reduced through pro-active planning at national and local levels. There is sufficient information to support the human and animal welfare benefits of including companion animals in national planning, and lessons learned through this and other experiences can assist planners in the development of comprehensive and locally relevant contingency plans.

## 1. Introduction

The chaotic atmosphere following disasters is an extremely charged environment both physically and emotionally for affected community residents, local governments as well as for incoming humanitarian and rescue teams [1,2]. Infrastructure is often affected at least to some degree and logistical difficulties in executing operations are expected. Supplies, equipment, medical treatments and facilities for people and animals, personnel, as well as funds to support response activities can be difficult to mobilize on short notice [2]. Despite the uncertainties and the immediate difficulties faced following these disasters, one thing we know for sure is that they will continue to occur, and are likely to increase due to environmental and climatic changes [3]. 

Large-scale disasters might make international news for a few minutes to weeks at the most, yet the effects on the ground last for years. Preparing for and responding to disasters is one of the greatest challenges facing the international community [4]. The economic, social, physical and emotional drain on societies is immeasurable and unfortunately a vast majority of disasters occur in low and middle income countries of the world in which resources to cope with large scale upheavals are limited [4,5]. Many lose family members or suffer the trauma of living through the event [6]. Others lose their homes, their possessions, their pets and their livestock, leaving them vulnerable and often with no livelihood [7]. It is critical to report on these events so that future responses are improved and tailored to environments and needs [1]; yet despite the fact that 85% of the disasters and 95% of all disaster-related deaths occur in the developing world, less than 1% of the disaster-related publications are originating from these countries [5].

Chile is one of the most seismically active countries in the world, with a long history of large-scale earthquakes, tsunamis and other disasters [8,9]. There is currently no national plan for companion animals following disasters. We report here on the difficulties encountered during a canine veterinary response following the 2010 earthquake and tsunami, and draw parallels to an earlier report of a human medical response in the 1985 earthquake. This report focuses on domestic dogs specifically because of these authors experiences gained while responding to a canine distemper virus (CDV) outbreak following the disaster; however domestic cats are of equal importance when considering companion animal planning. Using these examples from Chile, we demonstrate the importance of pre-planning and response coordination, and the value of reporting on the persistent difficulties to improve the response following disasters. 

## 2. Companion Animals in Disasters

The predominant literature available regarding animals and emergencies describes contingency planning in the event of outbreaks or introductions of disease with international trade importance [10]. Yet in recent years the inclusion of companion animals into national disaster preparedness plans has become more prevalent. This is in part due to studies conducted during or after disasters that have articulated the significant negative impacts on communities in the absence of plans that include household pets. For example, in 2001, Heath *et al.* identified risk factors for pet and owner evacuation [11,12]. Following Hurricane Katrina, Levy *et al.* [13] identified specific infectious disease risks in rescued cats and dogs, and Zottarelli and Hunt *et al.* studied the human psychological and emotional factors following the loss of their pets [14,15]. Risks of emergence of zoonotic and other infectious diseases following disasters have been reported by Ivers and Ryan [16], Ketai *et al.* [17], Wang *et al.* [18], Garde *et al.* [19,20] and Pasquali *et al.* [21], among others, all of which lend support to the mandatory inclusion of pets into disaster planning.

Despite this growing body of information, companion animals continue to be excluded from preparedness plans in the majority of countries. Plans are focused on saving human lives and at times organized animal rescue operations are forbidden to enter disaster areas [22]. Animals are at much higher risk than people because they are not the priority following disasters [7]. They are subject to abandonment or loss, a lack of food and water, absence or inadequate sheltering, injury, disease and entrapment, the magnitude of which depends on the location, severity of the disaster and availability of contingency plans and resources to cope [7,20]. Many of these negative effects can be mitigated with some advance planning.

**Figure 1:**
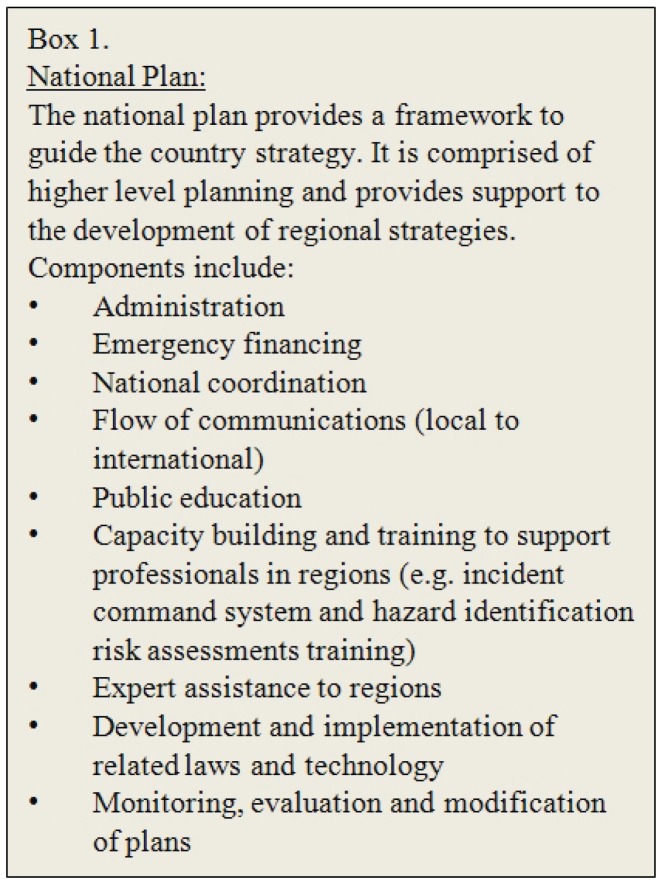
Description of the objectives of a national preparedness plan with examples of components.

**Figure 2:**
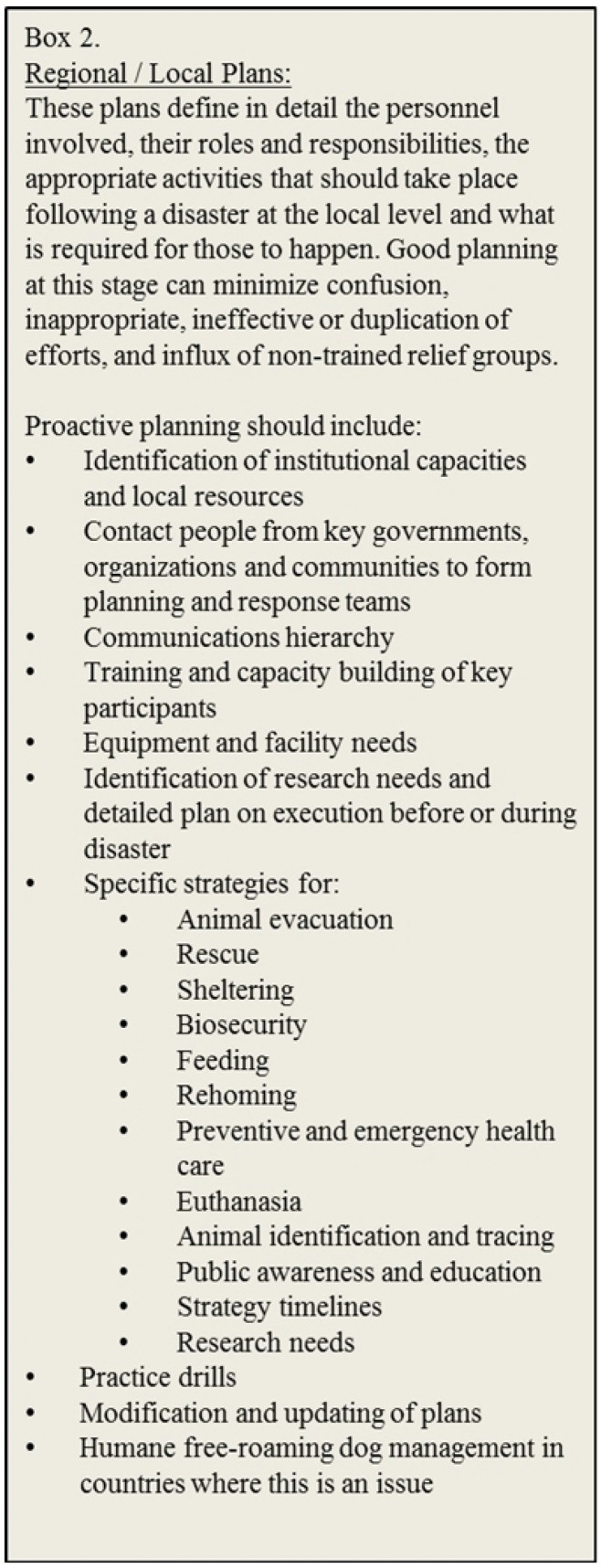
Description of the objectives of regional and local preparedness plans with examples of potential components.

**Figure 3:**
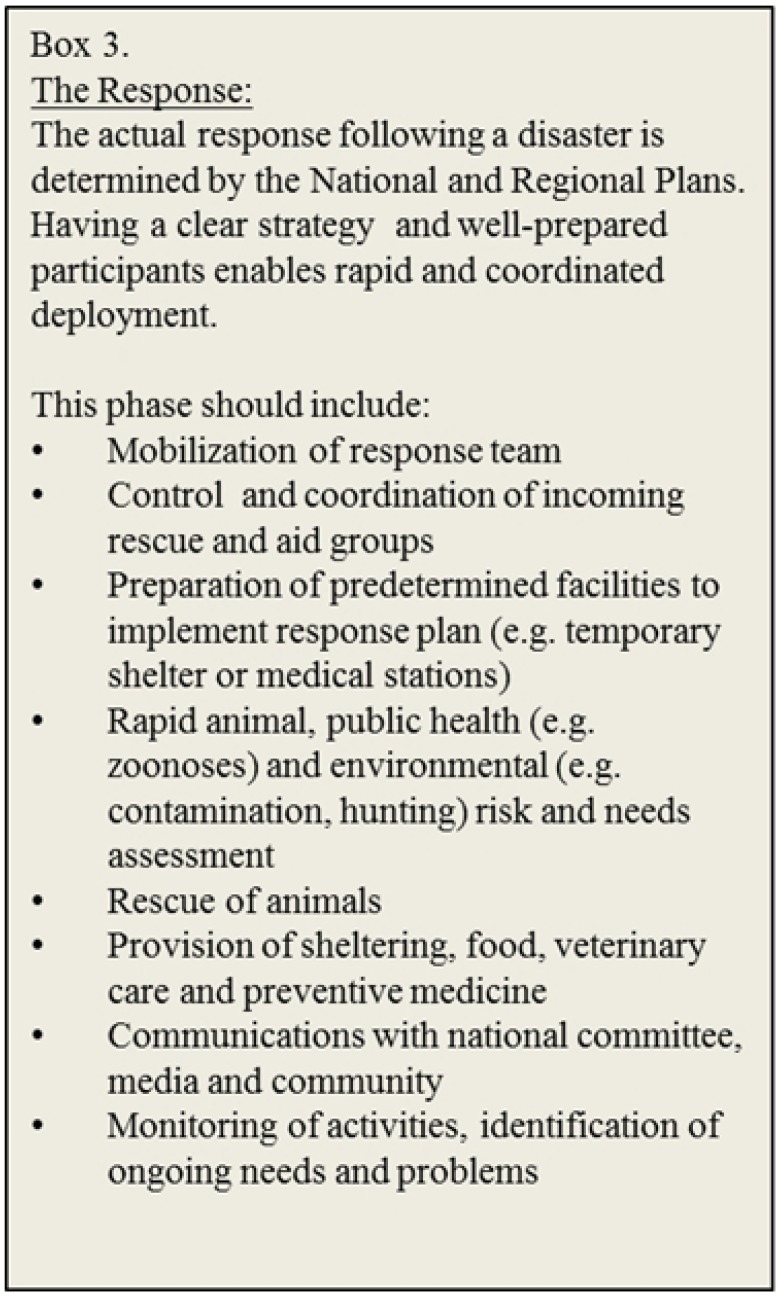
Description of the ground response immediately following a disaster with examples of typical operations.

**Figure 4:**
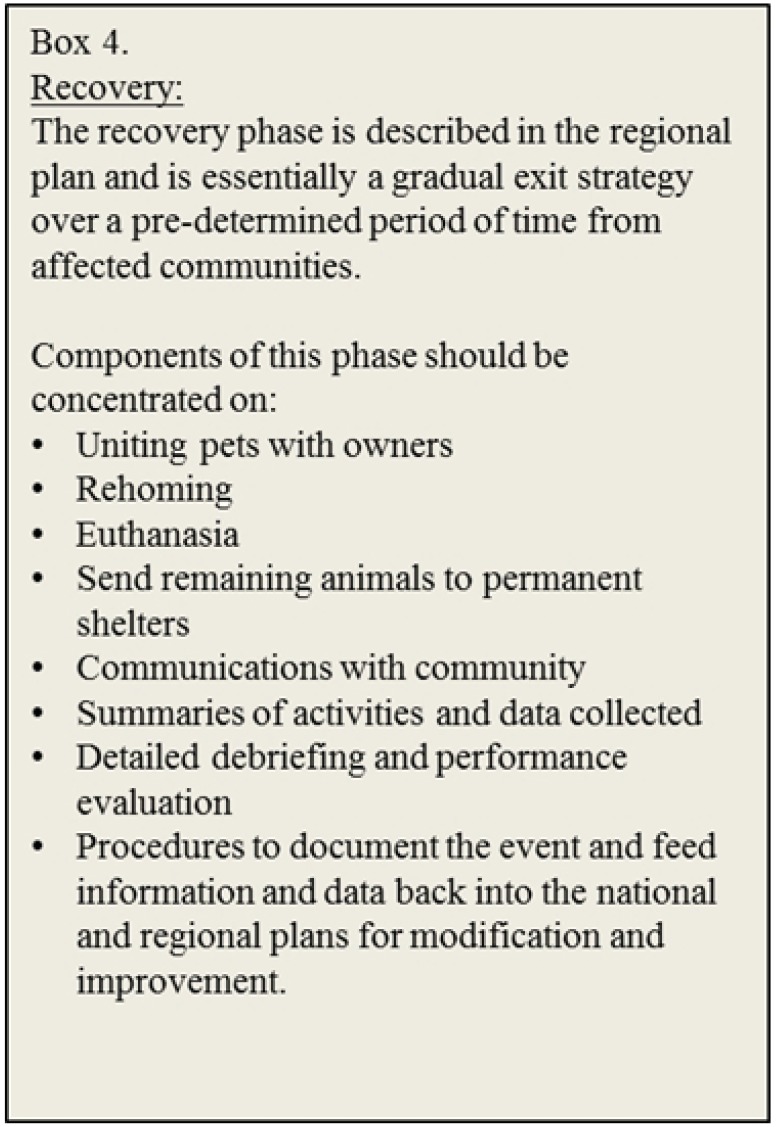
Description of the recovery period following the acute disaster phase with examples of typical activities.

Advances in our understanding of the impacts of disasters on people and their animals are critical as they provide the information needed to lobby for national legislation requiring the development of regional preparedness plans that include companion animals (e.g., PETS Act 2006) (Figure 1 and Figure 2). The result is proactive preparation for disasters and detailed planning of the response that is tailored to the location (Figure 3). There is a multitude of state and provincial plans from higher income countries available online, some including companion animals. However, there are few follow-up accounts evaluating the efficacy of these plans [1,23] or details of the long term recovery (Figure 4). This kind of information would serve to improve existing plans and offer guidance to those in the process of developing new preparedness strategies for companion animals. Furthermore, these available plans may only be relevant to “resource-rich” countries [5] where shelters, veterinary services, equipment, supplies and trained personnel are already available. In countries where many of these inherent services and facilities are absent, or the management of animals is dramatically different, disaster planning must reflect the culture, needs, resources and abilities of individual communities.

## 3. Data Collection During Disasters

Fortunately animal response teams are often quick to act following disasters. Yet, uncoordinated responses can delay well-intended operations, can render them less efficient and efforts can be duplicated or absent. No-one knows who is in charge, valuable information is lost, conflict arises, chaos is prolonged and accentuated, volunteers are confused and frustrated, and risks to residents and incoming assistants is continued even after the original disaster has passed (e.g., aftershocks, looting, food shortages) [1,24].

When responses are pre-planned and coordinated, residents and response officials alike realize their role in the recovery [2]. The majority of unharmed residents should be able to take care of themselves for a short time (e.g., 72 hours) so that officials can begin to implement the response despite the inevitable chaos [4,25]. Collection of information during the response and recovery phases allows planners to identify weaknesses in the existing response, evaluate and improve upon their plan and identify additional risks. Data generated provide a basis from which local governments as well as other regions and countries can learn from and improve or expand their own plans and responses. They also provide a basis for disaster research needs by identifying gaps in our knowledge or understanding. 

Simple but thorough medical records provide a summary of medical cases attended to during the disaster period and contribute to a needs assessment (e.g., medical supplies and biologics, diagnostic ability, capacity, funds) for future disaster planning. Incoming volunteers often lack experience in the recognition of endemic diseases, therefore record-keeping is of paramount importance in preventing misdiagnosis and inappropriate treatments [26,27]. Information collection from shelter inhabitants and other community members provides information on individual damages, pet loss or abandonment, livestock deaths, and ability to maintain original or new sources of income. This can be used as an indicator of community-wide effects of disaster, ongoing need for government or international aid and to identify and quantify unanticipated community/animal needs such as financial support, species specific sheltering, disease prevention or control and health care.

Unfortunately, these occasions to learn about the difficulties encountered in the immediate response are seldom documented and become lost opportunities for professionals of the health care and disaster contingency planning occupations. Although reporting on internal problems and inefficiencies may be interpreted as a failure, the information is extremely valuable for response improvement [1]. 

## 4. Chile: A Case Study

### 4.1. Medical Response—1985 Earthquake

On 3 March 1985, a 7.8 magnitude earthquake hit central Chile [8,28]. This earthquake was by no means the largest or most significant earthquake in Chile; however a paper published a year after the earthquake by the Chilean Ministry of Health details the response and the problems encountered by health professionals. This progressive and frank account describes the investigators’ objectives, that included collection of information about injuries, mortalities, and weekly reporting on the incidence of typhoid fever and hepatitis, and the obstacles encountered in meeting these goals. Although they emphasize that the immediate response by individuals was effective and generous, and medical professionals showed unbelievable abilities to adapt and deliver care under compromised conditions, they also experienced a host of problems that hindered their ability to accurately report on the disaster. The greatest problem identified was the lack of integrity of data collected (e.g., incomplete records and death certificates), misplacement of clinical files, inconsistency of information and data sources, and a general lack of understanding and appreciation of the importance of record-keeping [8]. Investigators identified a lack of interest in scientific investigation, a lack of reporting of experiences gained, and a general apathy and complacency observed as time lapsed. These greatly reduced their ability to accurately report on the post-disaster effects and maintain the momentum for ongoing research as time progressed [8].

### 4.2. Veterinary Response—2010 Earthquake

Twenty-five years later, on 27 February 2010, an 8.8 earthquake again rocked central Chile and was followed by a series of tsunami waves that destroyed numerous coastal towns [29]. Over 80% of the small town of Dichato was lost in the tsunami. Most of the initial government effort was logically focused on human care and restoration of essential infrastructure. A month following the earthquake, a CDV outbreak swept through dogs in Dichato and many were reported affected [20]. Veterinarians without Borders-Canada’s regional office from Valdivia, Chile together with veterinarians and student volunteers from the Universidad Austral de Chile and the Universidad de Concepción were asked by local animal welfare groups to assist with the objectives of disease diagnosis, containment and description and mitigation of the outbreak.

#### 4.2.1. Planning the Response

We consulted with other non-governmental organizations (NGOs) to gain an appreciation of the local situation and immediately solicited emergency disaster funds. A preliminary visit was planned to confirm the tentative diagnosis of CDV and to initiate a vaccination campaign in seven neighborhoods where the majority of the residents were housed or living in temporary camps. We formed four teams consisting of a veterinarian, one to two veterinary students to assist and restrain dogs and one record-keeper. Objectives of the field activity and study, along with the protocol were given in advance to all volunteers and team leaders. A biosecurity and safety information package as well as a veterinary supplies kit and cooler for vaccinations and blood samples was prepared and delivered to each team upon arrival on-site. Numbered medical records using a standardized format for recording physical exam findings were given to veterinarians and vaccination records were prepared for owners. A second visit repeating the protocols for physical exams and vaccination was conducted a month later.

#### 4.2.2. Animal Welfare Volunteers On-Site

There were many professionals, students and interested community members available to help with the campaign and study. Volunteers willingly exposed themselves to certain risks such as large aftershocks and looting, uncomfortable conditions and very long working hours. Nevertheless, much as the experiences documented by Reyes *et al.* [8], there were a number of factors that impeded our ability to work efficiently and effectively. There were numerous local ad hoc animal welfare groups and national and international NGOs that arrived in Dichato to offer assistance. In the absence of any national or regional preparedness planning for companion animals, there was no assigned coordinator from government, community or local NGOs to direct incoming rescue groups and therefore there was no harmonized response offered by animal health or welfare groups. Objectives of each group varied and included formal assessments, feeding of abandoned or homeless dogs, rehoming and provision of veterinary services such as vaccination, treatment of injuries and chronic conditions and surgical sterilization. However, many had no disaster or rescue training, they had very few resources to deal with the situation and some had no formal veterinary training being comprised of students and volunteers unlicensed to practice veterinary medicine. Few groups came prepared with food and water to support their own teams and lodging was difficult to secure.

Typical of post-disaster humanitarian efforts [8,30], the absence of a coordinated response among groups coupled with a lack of appropriate training and record-keeping, tended to complicate every subsequent veterinary effort as owners were unclear about what different groups had actually done or administered to their animals. There were no formal medical records generated by veterinarians, dogs were not marked when treated and owners were not given a record of treatments administered. Many owners thought their dogs had been vaccinated, when in fact they had been given antibiotic or anti-parasitic injections. There were reports alleging local animal rescue groups removed sick animals from Dichato to provide supportive care to them in other communities, which similar to the situation following Hurricane Katrina, introduces the risk of dissemination of diseases to new areas [13].

#### 4.2.3. Difficulties within the Team

Communications and pre-planning is complicated following disasters, and we experienced difficulties within our own veterinary teams. Internet and phone lines were only intermittently available which reduced our internal ability to plan site visits and confirm volunteer help. Public transportation and travel complications such as poor condition of highways, detours, and restricted access to gasoline complicated our travel plans. Personnel safety, lodging, food and water, and appropriate refrigeration of vaccines and blood samples also had to be accounted for while in Dichato which added to the logistical difficulties as many things were unavailable or very difficult to acquire.

Similar to the human medical response following the earthquake in 1985 [8], we experienced a loss of momentum over time, demonstrated by very poor turn-out rates of veterinarians and volunteers. Only 80% and 50% of our confirmed team members showed up to work in April and May, respectively. Reasons for poor turnout included other time commitments and priorities, cost of the trip, lack of interest and unknown reasons. 

There were also problems with the quality of reporting by veterinarians and assistants on the medical forms. Almost a quarter of the medical records were incompletely or incorrectly filled out in April in at least one or more of the physical exam categories, and much of the information on owners and animal history was incomplete. This dramatically reduced our group sample sizes when analyzing data following the campaign. We applied different forms of supervision and follow-up during the May visit which included daily meetings and frequent checks throughout the day to monitor protocols and procedures, and our numbers of incomplete medical records dropped from 22.5% to 12.1%. Although some level of chaos and reduced ability to perform is always expected following a natural disaster, we observed very similar and preventable difficulties within the veterinary response as with the medical response described during the 1985 earthquake [8].

#### 4.2.4. Local Support

Despite repeated attempts to contact, collaborate, register and coordinate our efforts during our visits to Dichato, we received no response from local governments regarding our activities. This is probably in part due to the immense burden of responsibility placed on these entities for restoration of infrastructure and human services, but is also indicative of a low value placed on the health and welfare of companion animals and the lack of appreciation for the potential for an increase in the multi-faceted problems that occur when dogs are abandoned or left to roam [19,20,31].

We also found that there was a much-reduced level of interest in the campaign by dog owners in May, accounting for our low vaccination rate on the second visit. We visited all temporary camps and the remaining intact neighbourhoods of Dichato for 3 days and simply could not solicit more interest in owners in any areas we visited to bring their dogs forward for a vaccination. This complacency was similar to findings reported by communities following the Chile earthquake in 1985 regarding a lack of interest in disaster efforts as time elapsed [8]. Without mandatory vaccination campaigns endorsed by local governments, the public interest in participating in a voluntary campaign did not appear to be sufficient to bring herd immunity to a protective level for diseases of high risk such as CDV [20].

## 5. Discussion and Conclusion

This brief report provides examples of the many challenges encountered during disaster responses, using Chile as an example. Similar to other Latin American countries, Chile has many free-roaming dogs (FRD), the majority of which are owned [19,20,21]. In this case, preparedness plans developed in other countries may not be relevant since the basic social structure (e.g., dog management, cultural values), the pre-existing infrastructure (e.g., animal-related legislation, shelters, veterinary clinics) and the resources available (e.g., medical supplies and equipment, trained personnel, national funds) may differ significantly between countries.

Fortunately, a response to assist animals in need was initiated immediately following this disaster. However, in the absence of a tailored companion animal plan for Chile, there was no organized care, treatment or sheltering for dogs and they were left to roam freely, aggravating the negative effects of having FRDs in non-disaster times and potentially providing a source for canine zoonoses to vulnerable in-contact residents [19,32,33]. The study of a CDV outbreak provided a valuable account of diseases that can occur in the animals themselves. However, there was no prior/baseline information about CDV prevalence from which to draw epidemiologic conclusions [20]. In the absence of government involvement and coordination, there were no constraints placed on the activities permitted by animal welfare groups or enforcement of non-veterinarians practicing medicine. There were no restrictions on movements of animals in and out of the area, posing risks of disease expansion when an infectious disease outbreak has been identified. There is also no legal requirement for pets to be permanently identified (e.g., microchip) and registered in a central registry to facilitate the control of dogs and cats and re-unification of pets with owners. Furthermore, disaster environments may not be the best location or time to be coordinating field sterilization campaigns due to the lack of hygiene, absence of provisioning of post-operative care, poor health and nutritional status of animals and lack of internal resources to cope with large groups of incoming volunteers. 

Chile is a country challenged with frequent disasters [8] and grave problems with free-roaming companion animals [34]. It is one of the 178 member countries of the World Health Organization for Animals (OIE) [35] and in theory would support the guidelines outlined in the Terrestrial Animal Health Code describing stray dog population control [36]. Although this document does not refer specifically to disasters, it does provide a comprehensive strategy for dealing with stray dogs where serious human, animal or welfare threats exist, with the objective of mitigating or preventing the myriad problems that can result when free-roaming dogs are abundant, for example following disasters.

Chile is also on the cusp of gaining international “developed” status [37] and could serve as a useful model for the rest of Latin America. However, there is in general, a serious lag time between the release of information about the need to incorporate companion animals into national planning and the legal mandate to do so. For example in 2008, Chile gained unfavorable international attention over its lack of inclusion of companion animals in the evacuation following the dramatic eruption of the Chaitén volcano [22]. In this case, fierce local and international lobbying prompted the government to mobilize the army to recover the abandoned dogs and it was reported that significant psycho-social and political damage would have been averted had companion animals been included in the planning process [9,22]. It is documented through worldwide experiences that human deaths increase when their pets are not included in disaster responses, that negative animal welfare effects and deaths are profoundly increased, and it is suggested that there could be an increase in the transmission of zoonotic disease transmission following disasters [7,19,20,21,22,38,39]. Unfortunately, lessons learned are not always incorporated into the cycle of evaluation and development or improvement of plans [1,22].

Based on available information and evidence, national and local authorities must take responsibility for the incorporation of companion animals into their planning process, instead of relying on international organizations that may be unfamiliar with the social, economic and cultural realities in which the disaster occurs, or on local NGOs that are often resource deficient and lack expertise [22]. As a form of monitoring and evaluation of field efforts and plan implementation, key leaders from NGOs and governments should conduct debriefing meetings to identify problems and successes encountered, and to report on those identified as a concrete step toward developing or improving existing response plans. Recommendations for future research should be driven by these reports generated following disasters. 

Studies of the veterinary disaster response are few, so lessons learned are extremely valuable. There is however, sufficient information in the literature to demonstrate the inherent links between companion animals, humans, livestock and wildlife health and well-being. Using a one-health philosophy, we must intuitively recognize the importance of managing people and animals together as part of a comprehensive disaster plan. 
